# A multicenter cross-sectional study on the correlation between lower urinary tract symptoms and sleep status in female nurses aged 40 years and above

**DOI:** 10.3389/fpubh.2026.1896771

**Published:** 2026-08-05

**Authors:** Xuecui Jiao, Chengshuai Zhang, Guodong Yang, Tingting Dong, Fude Li, Hongmei Zhang

**Affiliations:** 1 The Second People's Hospital of Dongying, Dongying, China; 2 Shengli Oilfield Central Hospital, Dongying, China

**Keywords:** cross-sectional study, female nurses, lower urinary tract symptoms, sleep, voiding behavior

## Abstract

**Objective:**

This multicenter cross-sectional study aimed to identify factors associated with lower urinary tract symptoms (LUTS) and to examine the relationship between LUTS, voiding behaviors, and sleep quality among female nurses aged ≥40 years in Shandong Province, China.

**Methods:**

Using a two-stage stratified cluster sampling design based on regional economic status, we selected 20 public tertiary hospitals and enrolled 2,455 eligible female nurses. LUTS were assessed using the validated Chinese version of the International Consultation on Incontinence Questionnaire-Female Lower Urinary Tract Symptoms (ICIQ-FLUTS), voiding behaviors with the Voiding Behavior Questionnaire, and sleep quality with the Pittsburgh Sleep Quality Index (PSQI). Multivariable binary logistic regression was used to identify factors associated with LUTS.

**Results:**

Age, BMI, cesarean section, vaginal delivery, constipation, diarrhea, daily fluid intake (≤500 mL) and coffee consumption were independently associated with LUTS (*p* < 0.05). Total LUTS score, total voiding behavior score and subdomain scores were positively correlated with PSQI score (*p* < 0.05). Hierarchical regression analysis showed that LUTS (yes/no), total voiding behavior score and the “urine holding” subdomain score entered the regression equation, collectively explaining 12% of the variance in sleep quality (*p* < 0.05).

**Conclusion:**

LUTS and maladaptive voiding behaviors, particularly habitual urine holding, are significantly correlated with poor sleep quality in female nurses aged ≥40 years. Occupational health interventions targeting modifiable voiding behaviors may offer a novel approach to improving sleep in this high-risk population. Future longitudinal studies are needed to establish causality.

## 1 Introduction

Lower urinary tract symptoms (LUTS) refer to a group of clinical symptoms associated with lower urinary tract dysfunction, including storage symptoms (e.g., frequency, urgency, nocturia), voiding symptoms (e.g., weak stream, straining) and post-voiding symptoms (e.g., incomplete emptying) (1). Affecting approximately half of adult women globally (2), LUTS represent a prevalent condition whose underlying pathophysiology remains incompletely understood. The prevalence of LUTS in women is increasing annually, seriously affecting physical and mental health, social function and quality of life. More than 60% of adults worldwide have at least one LUTS, and the prevalence is usually higher in women than in men. A Polish study reported that the prevalence of LUTS in the older adult(s) was 69.8%, with a particularly high rate in women (72.6%) (3). In China, a population-based survey reported that the prevalence of any LUTS among adult women was 46.5%, with storage symptoms being the most commonly reported (4). In Hong Kong, a study based on the International Prostate Symptom Score (IPSS) showed that 77.3% of women over 40 years had LUTS of varying degrees (5). Overall, although differences in population, ethnicity, region and cultural background cause some variation in LUTS prevalence worldwide, LUTS is a common condition globally.

As the largest segment of the clinical nursing workforce, female nurses are exposed to chronic occupational stressors including prolonged standing, extended work hours, and the psychological burden of caring for critically ill patients and managing distressed family members. These factors may lead to anxiety and excessive stress, affecting physical and mental health, and may also cause hormonal disturbances. Hormonal regulation is an important control mechanism, and studies have shown an association between changes in estrogen levels and LUTS (6). Female nurses exhibit a high prevalence of maladaptive voiding behaviors, particularly habitual urine holding, which may predispose them to LUTS (7). Age is an independent risk factor; the prevalence of LUTS increases with age. A multicenter study on LUTS in Chinese female nurses showed that the prevalence in those aged ≥40 years was significantly higher than in those <40 years, suggesting that age may be an important factor, but the LUTS status and related reasons in nurses aged ≥40 years need further analysis and verification (8).

Meanwhile, sleep is a common indicator of health status. Numerous studies have shown that clinical nurses generally have varying degrees of sleep problems. However, studies on the correlation between LUTS and sleep status in clinical nurses are rare. Therefore, this cross-sectional study aimed to investigate LUTS in female nurses and analyze its correlation with sleep quality, so as to provide a theoretical basis for improving the physical and mental health of female nurses.

## 2 Subjects and methods

### 2.1 Study subjects and data collection

Female nurses aged 40 years and above from 20 public tertiary hospitals in Shandong Province were enrolled between June and September 2024.

Inclusion criteria: (1) employment in a public tertiary hospital in Shandong Province; (2) female sex; (3) age ≥40 years; and (4) provision of written informed consent.

Exclusion criteria: (1) pregnancy, pursuing advanced studies, or being on leave during the study period; (2) three or more urinary tract infections within the past year (self-reported and confirmed by medical records where available); (3) lower urinary tract injury or pelvic/urogenital surgery within the preceding 3 months; (4) active cancer chemotherapy; (5) spinal cord injury, cerebrovascular disease, or renal disease; or (6) use of diuretics during the study period.

Ethics approval and informed consent: The study was approved by the Ethics Committee of Shengli Oilfield Central Hospital. All participants provided written informed consent prior to enrollment and were informed of their right to withdraw at any time without consequence.

### 2.2 Sampling method and sample size

This study is part of the Shandong Female Nurses’ Lower Urinary Tract Health Promotion Project, which is affiliated with the Nurse Urinary Related Health Study (NURS) (8). Electronic questionnaires were used to collect data via a secure online platform. To ensure data quality, two trained staff members in each sample hospital assisted in data collection and verified questionnaire completeness. A two-stage cluster sampling design was adopted.

Stage 1 (Hospital selection): Based on the interquartile range of gross domestic product (GDP) in 2023, the 16 prefecture-level cities in Shandong Province were divided into four economic regions: high, medium-high, medium-low and low. The number of tertiary hospitals in each region was calculated, and 20% of the hospitals were selected using a random number table combined with convenience sampling. A total of 20 hospitals were selected.

Stage 2 (Participant enrollment): All nurses meeting the inclusion/exclusion criteria in the selected hospitals were surveyed by cluster sampling.

Sample size calculation: Based on a previous study reporting a 51.1% prevalence of LUTS in female nurses (8), with a 95% confidence level, a desired absolute precision of 3%, a design effect of 1.5 for cluster sampling, and accounting for a 20% non-response rate, the minimum required sample size was calculated to be approximately 1,800. We ultimately enrolled 2,455 participants, exceeding the requirement.

### 2.3 Research instruments

#### 2.3.1 Demographic and health status questionnaire

A self-designed questionnaire collected information on age, professional title, years of work, smoking, alcohol consumption, fluid intake, medication use, pregnancy and delivery history (including number of vaginal deliveries and cesarean sections), disease history (including constipation, diarrhea, and other chronic conditions), and exercise duration, frequency and intensity. Menopausal status was also recorded.

#### 2.3.2 Female Lower Urinary Tract Symptoms Questionnaire

The validated Chinese version of the International Consultation on Incontinence Questionnaire-Female Lower Urinary Tract Symptoms (ICIQ-FLUTS) was used to assess LUTS over the past 4 weeks (8). The questionnaire consists of 12 items covering three domains: storage symptoms (items 1–4, score range 0–16), voiding symptoms (items 5–7, score range 0–12), and incontinence symptoms (items 8–12, score range 0–20). For item 4 (frequency), the response options were scored as follows: options 0–3 were assigned 0 points, options 4 and 5 were assigned 1 point, option 6 was assigned 2 points, option 7 was assigned 3 points, and options 8 and 9 were assigned 4 points. For each individual item, a score ≥2 was defined as having that specific symptom. LUTS was defined as present if the participant scored ≥2 on at least one of the 11 symptom items (items 1–8 and 10–12; item 9 was excluded as it assesses impact rather than symptom presence). Both the binary LUTS status and the continuous total score were used in analyses. Higher scores indicate more severe symptoms. The Cronbach’s *α* for the Chinese version was 0.87 in this study (8).

#### 2.3.3 Voiding Behavior Questionnaire

Developed by Chinese scholar Wang Kefang (7), the Voiding Behavior Questionnaire includes 19 items across five dimensions: place of urination, urination without urge, urine holding, straining to urinate, and urination posture. Excluding items 12 and 13, higher scores (range 0–76) indicate more maladaptive voiding behaviors. The Cronbach’s *α* for the total scale in this study was 0.89.

#### 2.3.4 Pittsburgh Sleep Quality Index

Developed by Buysse and sinicized by Liu Xianchen et al. (Cronbach’s α = 0.83–0.94, item reliability >0.97) (9). A total score >7 indicates sleep disturbance.

### 2.4 Statistical analysis

SPSS 23.0 and R (version 4.2.2) were used. A two-sided *p* < 0.05 was considered statistically significant. Categorical data were expressed as frequencies and percentages; continuous data as mean ± standard deviation.

To identify factors independently associated with LUTS while accounting for potential confounding, we conducted a multivariable binary logistic regression. Model fit was assessed using the Hosmer-Lemeshow test and Nagelkerke *R*^2^. Multicollinearity was assessed using variance inflation factors (VIF < 5 for all variables in the final model).

## 3 Results

### 3.1 General characteristics and factors associated with LUTS

Of the 2,665 eligible nurses invited to participate, 2,455 completed the survey. Of these, 1,448 (59.0%) had LUTS. The mean PSQI score was 5.85 ± 3.45.

The characteristics of participants by LUTS status are shown in Table 1. Bivariate analysis revealed that age, BMI, cesarean section, vaginal delivery, constipation, diarrhea, fluid intake and coffee consumption were significantly associated with LUTS.

**Table 1:** Participant characteristics by LUTS status (*N* = 2,455).

Characteristic	LUTS (Yes) (*n* = 1,448)	LUTS (No) (*n* = 1,007)	Statistic (t/χ^2^)	*P*-value
Continuous variables	Mean ± SD	Mean ± SD		
Age (years)	46.8 ± 4.9	44.3 ± 4.2	t = 13.20	<0.001
BMI (kg/m^2^)	23.54 ± 2.77	23.15 ± 2.57	t = 3.55	<0.001
Categorical variables	*n* (%)	*n* (%)		
Cesarean section			χ^2^ = 12.77	<0.001
Yes	563 (38.9)	464 (46.1)		
No	883 (61.1)	543 (53.9)		
Vaginal delivery			χ^2^ = 22.96	<0.001
Yes	906 (62.6)	536 (53.2)		
No	538 (37.4)	471 (46.8)		
Constipation			χ^2^ = 43.61	<0.001
Yes	440 (30.4)	187 (18.6)		
No	1,008 (69.6)	820 (81.4)		
Diarrhea			χ^2^ = 8.79	0.003
Yes	66 (4.6)	23 (2.3)		
No	1,382 (95.4)	984 (97.7)		
Daily fluid intake			χ^2^ = 18.52	<0.001
≤500 mL	147 (10.2)	70 (7.0)		
501–1,500 mL	721 (49.8)	465 (46.2)		
1,501–2,500 mL	433 (29.9)	367 (36.4)		
≥2,501 mL	147 (10.2)	105 (10.4)		
Coffee consumption			χ^2^ = 10.46	0.034
Never	988 (68.2)	676 (67.1)		
1–3 times/month	338 (23.3)	213 (21.2)		
1–2 times/week	66 (4.6)	56 (5.6)		
≥3 times/week	56 (3.9)	62 (6.2)		

To identify factors independently associated with LUTS while adjusting for potential confounders, we performed multivariable binary logistic regression including all covariates listed in Table 1. As shown in Table 2, age, BMI, vaginal delivery, constipation, diarrhea, daily fluid intake ≤500 mL, and coffee consumption ≥3 times/week remained independently associated with higher odds of LUTS. By contrast, cesarean section was associated with lower odds of LUTS. All associations were statistically significant (all *p* < 0.05). The Hosmer-Lemeshow test indicated good model fit (χ^2^ = 8.32, *p* = 0.402), and the model explained approximately 18% of the variance in LUTS.

**Table 2:** Multivariable logistic regression of factors associated with LUTS.

Variable	OR	95% CI	*P*-value	VIF
Age (per 1-year increase)	1.12	(1.09–1.15)	<0.001	1.08
BMI (per 1 kg/m^2^ increase)	1.06	(1.03–1.09)	<0.001	1.05
Cesarean section (Yes vs. No)	0.75	(0.63–0.89)	<0.001	1.12
Vaginal delivery (Yes vs. No)	1.48	(1.24–1.77)	<0.001	1.15
Constipation (Yes vs. No)	1.67	(1.36–2.05)	<0.001	1.06
Diarrhea (Yes vs. No)	1.75	(1.07–2.86)	0.025	1.03
Daily fluid intake ≤500 mL (Yes vs. No)*	1.40	(1.03–1.91)	0.032	1.04
Coffee consumption (≥3/week vs. Never)	1.54	(1.01–2.36)	0.045	1.04

Reference group: daily fluid intake >500 mL. Model fit: Hosmer-Lemeshow χ^2^ = 8.32, *P* = 0.402; Nagelkerke *R*^2^ = 0.18.

### 3.2 Association between voiding behavior and sleep disturbance

Participants with sleep disturbance (PSQI >7) had significantly higher total voiding behavior scores than those without (Table 3).

**Table 3:** Comparison of voiding behavior scores by sleep disturbance status.

Variable	Sleep disturbance (PSQI>7)	*t*-value	*P*-value
Total voiding behavior score	Yes (*n* = 865): 48.15 ± 10.51	8.49	<0.001
	No (*n* = 1,590): 44.24 ± 11.08		

### 3.3 Correlation between voiding behaviors and sleep status

PSQI score was positively correlated with all dimensions of voiding behavior (all *p* < 0.01; Table 4). The strongest correlation was observed between PSQI and the “urine holding” subdomain (r = 0.26, *p* < 0.01). The total voiding behavior score also showed a moderate positive correlation with PSQI (r = 0.25, *p* < 0.01).

**Table 4:** Pearson correlation matrix between voiding behavior subdomains and PSQI score.

Variable	Place	Non-urge voiding	Holding	Straining	Posture	Total behavior
PSQI	0.13**	0.16**	0.26**	0.18**	0.13**	0.25**

** *p* < 0.00 (two-tailed). Bold indicates the strongest correlation.

### 3.4 Association between LUTS and sleep quality

The PSQI score in the LUTS group (6.61 ± 3.50) was significantly higher than that in the non-LUTS group (4.74 ± 3.07; *t* = 14.00, *p* < 0.001). In the LUTS group, 633 (43.7%) had sleep disturbance (PSQI>7), compared to 232 (23.0%) in the non-LUTS group (χ^2^ = 111.23, *p* < 0.001) (Table 5).

**Table 5:** Sleep quality comparison between LUTS and non-LUTS groups.

Variable	LUTS (*n* = 1,448)	Non-LUTS (*n* = 1,007)	Statistic	*P*-value
PSQI score (mean ± SD)	6.61 ± 3.50	4.74 ± 3.07	*t* = 14.00	<0.001
Sleep disturbance (PSQI>7), *n* (%)	633 (43.7)	232 (23.0)	χ^2^ = 111.23	<0.001

### 3.5 Regression analysis of LUTS and voiding behaviors on sleep quality

Regression analysis was conducted with PSQI total score as the dependent variable. Model 1 included LUTS (yes/no) and total voiding behavior score; Model 2 included the five voiding behavior subdomains separately. Results showed that LUTS (yes/no), total voiding behavior score (Model 1), and the “urine holding,” “urinating without desire,” and “voiding with force” subdomains (Model 2) were significantly associated with PSQI score (all *p* < 0.001). Model 1 explained 11% of the variance in sleep quality (*R*^2^ = 0.11, *p* < 0.001), and Model 2 explained 8% (*R*^2^ = 0.08, *p* < 0.001) (Tables 6, 7). All VIF values were below 2, indicating no significant collinearity. The intraclass correlation coefficient (ICC) for hospital-level clustering was 0.03, indicating minimal clustering effect.

**Table 6:** Regression analysis of factors associated with PSQI score (Model 1).

Variable	Beta	B (95% CI)*	*P*-value	VIF
LUTS (Yes vs. No)	0.22	1.38 (1.10, 1.66)	<0.001	1.06
Total voiding behavior score	0.20	0.07 (0.05, 0.09)	<0.001	1.06

*R*^2^ = 0.11, *P* < 0.001.

**Table 7:** Regression analysis of factors associated with PSQI score (Model 2 – voiding behavior subdomains).

Variable	Beta	B (95% CI)*	*P*-value	VIF
Urine holding	0.20	0.11 (0.09, 0.13)	<0.001	1.28
Urinating without desire	0.08	0.03 (0.01, 0.05)	<0.001	1.18
Voiding with force	0.08	0.02 (0.01, 0.04)	<0.001	1.26
Place of urination	0.00	0.00 (−0.15, 0.15)	0.808	1.25
Position of urination	0.03	0.05 (−0.03, 0.13)	0.189	1.20

Model *R*^2^ = 0.08, *P* < 0.001.

## 4 Discussion

### 4.1 Factors associated with LUTS in female nurses aged ≥40 years

The observed LUTS prevalence of 59.0% exceeds the 51.1% reported among Chinese female nurses of all ages (8) and is also higher than rates documented in community-dwelling women in Korea (54.2%) (10) and the international EPIC study (46% in women) (2). This high prevalence likely reflects the combined effects of an aging population (all ≥40 years) and the unique occupational stressors of nursing, such as high stress, prolonged standing, and frequent urine holding.

In the multivariable analysis, age and BMI were confirmed as independent correlates of LUTS. The age-related increase in LUTS prevalence likely reflects age-associated declines in pelvic floor muscle strength and changes in bladder sensory function (11). Elevated BMI may contribute through increased intra-abdominal pressure and chronic mechanical stress on pelvic floor supports (12). Prospective cohort studies have further confirmed that higher BMI is a significant predictor of urinary incontinence in women (13), and a meta-analysis of Chinese women demonstrated a consistent association between obesity and stress urinary incontinence (14).

The independent association with vaginal delivery is consistent with the hypothesis that vaginal childbirth causes direct trauma to pelvic floor musculature and innervation (15). Interestingly, cesarean section was associated with lower odds of LUTS (OR = 0.75), although its protective effect may diminish over time (16). Findings from a large Norwegian cohort study corroborate this pattern, showing that the protective effect of cesarean section against urinary incontinence is most pronounced in the short term and attenuates with advancing age (17).

Constipation and diarrhea were both independently associated with LUTS. The shared pelvic innervation of the bladder and rectum provides a plausible anatomical basis for this link (18). A growing body of evidence suggests that this symptom overlap may reflect common neurobiological pathways involving pelvic autonomic nerve dysfunction (19). Chronic constipation may also lead to straining, which can further weaken pelvic floor support.

Notably, low daily fluid intake (≤500 mL) was a significant factor. While it might seem counterintuitive, concentrated urine can chemically irritate the bladder mucosa and increase the risk of urinary tract infections, potentially exacerbating LUTS (20). Similar findings have been reported in cross-sectional studies of women (21), and clinical reviews have emphasized that both inadequate and excessive fluid intake may have adverse effects on bladder health (22). Finally, frequent coffee consumption was associated with LUTS, likely due to caffeine’s diuretic effects and ability to increase detrusor excitability (23). Population-based studies have similarly identified coffee consumption as a risk factor for urinary incontinence in women, particularly among those consuming more than two cups per day (24).

### 4.2 Correlation between voiding behavior and sleep status

A key finding of this study is the strong positive correlation between maladaptive voiding behaviors and poor sleep quality. Nurses with sleep disturbance had significantly higher total voiding behavior scores. The “urine holding” dimension showed the strongest correlation with PSQI (r = 0.26), suggesting that this is a critical behavior linking occupational constraints to poor sleep. Habitual urine holding may promote detrusor overactivity, which can in turn exacerbate nocturia—a primary contributor to sleep fragmentation (25). Longitudinal studies have shown that habitual voiding patterns established over years may contribute to the development of irreversible bladder dysfunction, underscoring the importance of early behavioral intervention (26). Furthermore, the correlation between “voiding with force” and worse sleep suggests that obstructive or underactive bladder symptoms also contribute to nocturnal awakenings (27).

### 4.3 Correlation between LUTS and sleep status

The association between LUTS and poor sleep was robust. The prevalence of sleep disturbance in the LUTS group (43.7%) was nearly double that of the non-LUTS group (23.0%). In the regression model, the presence of LUTS alone contributed significantly to the variance in PSQI scores.

We hypothesize a bidirectional relationship between LUTS and sleep disturbance. Nocturia and urgency directly fragment sleep, while sleep deprivation may lower bladder sensory thresholds and impair pelvic floor muscle recovery, potentially exacerbating LUTS (28). This potential vicious cycle underscores the need for integrated interventions that address both LUTS and sleep hygiene.

### 4.4 Interpretation of the explained variance

Although LUTS and voiding behaviors were significant correlates of sleep quality in the regression models, they collectively explained only 12% of the variance in PSQI scores. This modest effect size suggests that other unmeasured factors—likely including shift work patterns, menopausal status, anxiety and depression, and work-related stress—play substantial roles in determining sleep quality in this population. A recent predictive model based on the Chinese Nurse Health Cohort identified work-related stress and shift schedules as the strongest predictors of sleep disturbance among nurses (29). Additionally, anxiety has been shown to be associated with the relationship between nocturia and sleep quality in older women, suggesting that psychological factors may be important confounders (30). Clinically, this indicates that while addressing LUTS and voiding behaviors may be a useful component of sleep improvement interventions, comprehensive approaches addressing multiple sleep determinants are needed.

### 4.5 Implications for occupational health interventions

Our findings suggest several practical implications for occupational health programs targeting female nurses.

First, healthcare institutions should incorporate LUTS screening into routine occupational health assessments, particularly for nurses aged ≥40 years. Second, educational programs promoting healthy voiding behaviors—including regular voiding schedules, avoidance of prolonged urine holding, and adequate daily fluid intake (>500 mL)—should be implemented. Third, workplace modifications ensuring reasonable access to bathroom facilities during shifts are essential. Fourth, given the modest contribution of LUTS to sleep quality variance (12%), sleep improvement strategies should adopt a multifaceted approach that includes LUTS management alongside other sleep determinants such as shift scheduling and stress reduction.

### 4.6 Strengths and limitations

The major strengths of this study include its large, multi-center sample, rigorous stratified cluster sampling, and use of validated instruments. The application to account for hospital-level clustering further strengthens the validity of our findings.

However, several limitations must be acknowledged. First, the cross-sectional design prevents us from establishing causality. Second, we did not assess other important potential confounders such as shift work type, anxiety/depression, menopausal status, or family support, which may influence sleep quality. Third, the reliance on self-reported questionnaires may introduce recall and social desirability biases, although the use of validated instruments mitigates this concern to some extent. Fourth, our LUTS definition (≥2 on any symptom item) may have included mild cases with limited clinical significance; future studies should consider sensitivity analyses using higher thresholds. Future longitudinal studies are needed to confirm our findings and establish causal pathways.

## 5 Conclusion

This large multicenter study reveals a high prevalence of LUTS (59%) among female nurses aged ≥40 years in Shandong Province. Multiple factors, including age, BMI, history of vaginal delivery, constipation, diarrhea, low fluid intake, and coffee consumption, are independently associated with LUTS. Critically, LUTS and maladaptive voiding behaviors, particularly habitual urine holding, are significantly correlated with poor sleep quality and collectively explain 12% of its variance.

These findings suggest that occupational health programs for nurses should: (1) incorporate education on healthy voiding behaviors, including regular voiding schedules and adequate fluid intake; (2) address modifiable lifestyle factors such as coffee consumption; (3) ensure adequate bathroom access during shifts; and (4) screen for LUTS as part of routine occupational health assessments, particularly in nurses aged ≥40 years.

Future longitudinal studies are needed to establish causal pathways, and intervention trials should test whether modifying voiding behaviors improves both LUTS and sleep outcomes in this high-risk occupational group.

## Data Availability

The raw data supporting the conclusions of this article will be made available by the authors, without undue reservation.
